# Suffering, authenticity, and meaning in life: Toward an integrated conceptualization of well-being

**DOI:** 10.3389/fpsyg.2022.1079032

**Published:** 2022-12-06

**Authors:** Wojciech Kaftanski, Jeffrey Hanson

**Affiliations:** Human Flourishing Program, Harvard University, Cambridge, MA, United States

**Keywords:** suffering, authentic (authenticity), meaning in life and well-being, well-being, integrated conceptualization of well-being, Søren A. Kierkegaard, subjective well-being, objective well-being

## Abstract

Most conceptions of well-being either ignore suffering or assume an ideal version of human life in which suffering would be eliminated. This trend is especially emblematic of positive psychology. Recent research on well-being indicates a mediating function of meaning in life between suffering and well-being demonstrating that making sense of past experiences is significantly correlated with high presence of meaning in life. Hence, meaning-making serves the role of an active coping mechanism that alleviates suffering. This and related strategies of defining, measuring, and augmenting well-being however overlook a form of suffering that is ineliminable and in fact essential to personal growth. In this paper the insights of the existentialist philosopher Søren Kierkegaard are developed to formulate an integrated conceptualization of well-being that regards “negative” affects as crucial for a rich and complete life. The complexity of the relationship between meaning in life, suffering, and authenticity concerning well-being are discussed. A synthetic perspective on the subjective dimension of the experience of suffering and on the objective nature of human limitations that often cause suffering is discussed in relation to the notions of meaning in life and authenticity. Finally, an integrated conceptualization of well-being is posited. It entails suffering as constitutive of meaning in life and authenticity, which are key components of a well-lived life.

## Introduction

Dominant scientific conceptions of well-being either ignore suffering or treat it as an impediment to a well-lived life (Quick and Henderson, 2016; CDC, 2018; Simons and Baldwin, 2021; Hofmann, 2022; WHO, 2022). They represent what Fowers et al. (2017) call “The modern project to reduce suffering,” emblematic of the goals of positive psychology. Cassell (2004) defines the nature of suffering in relation to injury that causes different levels of the destruction of a person. It can be experienced before, during, and after an illness. Seligman (2012a) presents the prescriptive rule to “minimize our misery” as key to well-being. This preventive imperative of well-being has its counterpart in the famous *PERMA* model, which comprises positive emotion, engagement, (positive) relationships, meaning, and accomplishment (Seligman, 2018). These components that comprise *PERMA* can and should be pursued for their own sake; they are defined and measured independently from each other (Seligman, 2012a). Except for positive emotions, the development of the remaining four components of well-being can be measured by the growth of positive emotions around them. For instance, a good feeling about one’s relationships is a sign of good-standing or improvement in that area of well-being. Seligman (2012b) famously states delivering The Tanner Lectures on Human Values, “We should be just as concerned with making the lives of people *fulfilling* as we are with *healing pathology*” (Seligman, 2012b, p. 233). “None of the five elements show how to constructively approach suffering, dependency, frailty, or weakness,” bemoan Fowers et al. (2017) in their *Frailty, Suffering, and Vice: Flourishing in the Face of Human Limitations*.

Not all suffering is a sign of pathology (Wong, 2017; Wong and Bowers, 2018). Wong’s (2011) prominent manifest, “Positive Psychology 2.0,” calls for a reformulation of positive psychology to constructively integrate suffering in well-being by balancing well-being’s focus on positive and negative emotions: “psychology of well-being needs to study both the perils of happiness and the benefits of suffering” (p. 75). This intuition agrees with Haybron (2017) claim “A crucial task for any theory of well-being is to give a credible accounting of the value of pleasant and unpleasant experiences, especially suffering” (p. 12). In the spirit of humanistic-existential positive psychology that draws on figures such Victor Frankl and Rollo May (Wong et al., 2021), this article draws on the psychological-existential thought of the Danish nineteenth-century religious thinker Søren Kierkegaard. Provided analysis of Kierkegaard’s view of meaning in life, authenticity, and suffering is used to present an integrated conceptualization of well-being treats suffering as ineliminable part of human life essential to a well-lived life.

Kierkegaard is largely on a suspicious track regarding conceptualization of well-being as oriented toward minimizing suffering and maximizing positive emotions across numerous domains of well-being. Not all suffering and negative emotions around it signal diminished well-being in individual or communal lives. Hence, maximizing positive emotions or minimizing those that are negative should not be understood as a blanket theory for having a well-lived life. For Kierkegaard there are forms of necessary suffering that are not pathological in nature (Hanson, 2021a). Existential suffering is a hallmark of the complexity of human spiritual-psychological composition (Hanson, 2021b). Humans experience anxiety and despair because spiritual beings have spiritual needs, for Kierkegaard. “Even the struggling, suffering religious believer is on the right path; his or her sufferings in fact indicate that he or she is on the right path,” comments Watkin (2001, p. 8) on Kierkegaard’s existential psychology of suffering. Anxiety and despair are important for developing a holistic life project that largely includes indicators of well-being identified by Seligman, such as relations, meaning, or achievement. Instead of normatively qualifying individual relations or achievements as “positive,” Kierkegaard would suggest striving for “meaningful” relations or achievements in a well-lived life. And it is suffering that in many cases allows individuals to see their relations or achievements as “meaningful.” The other point of contention is how well-being is defined and how it can be measured. While Seligman is no stranger to objective definitions and measures of well-being across various domains, together with a vast majority of positive psychologists, he prioritizes subjective well-being in his research. This is precisely what Kierkegaard finds problematic in conceptualizing and measuring well-being. Kierkegaard stresses that we may be mistaken about the state of our well-being because we may simply lack relevant knowledge about the goods important to our happiness but also the role of negative affects and negative occurrences in the formation of our existential projects (Kierkegaard, 1980). Humans also can be victims of self-deception and willful blindness. It is unsurprising that people shy away from learning about the true state of their existences. Bypassing or coping with negative emotions may be in fact an impediment to one’s well-being as it diverts one from the lack of the eternal in their lives (Bernier, 2015).

## Meaning in life and suffering

Meaning is an important quality that humans attribute to such different elements in the world as natural disasters, art, relationships, financial assets, but also to sentences, judgments, values, and nature. Meaning is considered to be a great motivator in such different fields as public health, work, education, politics, and leadership (Morrison et al., 2007). Experiencing meaning is an important contributor to well-being and health (Steger, 2009). In relation to life, philosophers and psychologists developed theories of meaning in life (which often have an individual dimension) and the meaning of life (that look at life in a more general sense; Battista and Almond, 1973; Cottingham, 2003; Glaw et al., 2016). A widely accepted tripartite definition of meaning in life comprises “purpose (having goals to work toward or finding benefits from a specific event), significance (a sense of feeling value or mattering), and coherence (the feeling that the world and one’s experiences make sense)” (Edwards and van Tongeren, 2020, p. 722; *Cf.* Heintzelman and King, 2013; Martela and Steger, 2016).

Psychological literature treats suffering as an impediment to human well-being. Cassel’s definition of suffering is emblematic of that trend:

Suffering occurs when persons perceive their impending destruction or loss of integrity as persons, and continues until the threat of disintegration is passed or until the integrity of the person can be restored in some other manner. Most generally, then, suffering can be defined as the state of severe distress associated with events that threaten the intactness of persons (Cassell, 2002, p. 514).

This definition is paradigmatic of the treatment of suffering in public health; it stipulates that suffering be minimized or eliminated to secure or increase individual well-being. Indeed, mainstream positive psychology tends to take as its therapeutic goal maximization of positive affect and minimization of suffering often through coping mechanisms (Fredrickson, 2009; Bolier et al., 2013). This approach is especially entrenched in views that belong to preventive methods in mental-health and well-being in education (Conley et al., 2015; Bettis et al., 2017), the work-place (Elkin and Rosch, 1990; LaMontagne et al., 2007), and general public health (Stjernswärd et al., 2007). Important work has been published on the positive role of meaning in coping with suffering (Davis et al., 1998; Park, 2008; Park et al., 2008).

Study by Edwards and van Tongeren (2020) indicates a mediating function of meaning in life between suffering and well-being. Their research suggests that especially past suffering—rather than present suffering—is highly correlated with high presence of meaning in life. Suffering, if the meaning of which can be established, contributes to one’s sense of well-being; meaning in life mediates between suffering and well-being. While an important step toward reimagining the role of suffering for well-being, Edwards & van Tongeren’s study conceptualizes well-being as “satisfaction with life;” hence it prioritizes the role of self-reporting in defining and benchmarking well-being. It also implies meaning-making to serve in the role of an active coping mechanism that effectively deals with suffering. This and related strategies of defining, measuring, and augmenting well-being however overlook a form of suffering that is inevitable and in fact critical to personal development insofar as it propels individuals toward their envisioned ideal of a well-lived life that is both individual and socially constructed.

Recent research on meaning in life and authenticity that draws on Kierkegaard suggests the importance of “negative” affects for a rich and complete life (Hanson, 2021a,b). For Kierkegaard, meaning in life requires a form of necessary suffering, because the meaning of life derives from the individual’s lifelong effort to attain their own ideal version (Hanson, 2021a). Suffering so understood is aligned with a view that suffering can be “transformative”(Sacks, 2007; Fowers et al., 2017); on the one hand, it can be transformed into something good (not necessarily positive); on the other, suffering allows one to be opened to a formative transformation. Especially the latter position on suffering is strongly aligned with Kierkegaard’s project of individual perfection developed throughout much of his authorship (Kaftanski, 2021a, 2022), yet formulated most strongly in relation to meaning in life in *Practice in Christianity*. In that book, Kierkegaard introduces a model that represents life development that requires construction of an individual’s “image of perfection” that then must be translated into actual life (Kierkegaard, 1991). The grandeur of the ideal image of oneself depends on the force of one’s imaginative powers; yet the faculty of the imagination cannot itself translate that image into reality (Kaftanski, 2021b, 2022). If one could experience their life in imagination just as they can and should experience it in actuality, Kierkegaard indicates, “then there would be no meaning in life” (Kierkegaard, 1991, p. 188). Meaning in life requires this creative tension between possibility and actuality. Yet the attainment of meaning is marked by a necessary suffering that stems from the fact that one’s imagined ideal self and the actual self are not the same. “We do not merely suffer because of what happens to us. We also suffer from the imaginative construction that we have brought about ourselves,” as Rosfort (2015, p. 463) summarizes the suffering-generating tension between the imagined and actual selves.

Striving to realize the ideal of one’s project of existence is marked by suffering. While this paper builds a positive vision of suffering in relation to despair and a project of individual becoming, it is important to indicate that Kierkegaard acknowledges forms of despair that are pathological (Theunissen, 2005; Rosfort, 2015). Indeed, suffering caused by despair is meant to be ultimately eliminated by love and faith. Despair is the condition whereby an individual does not want to be herself or desires to be someone else. These can be caused and maintained by trauma, social comparison, resistance to seeking help, defiance in relation to the transcendent, or clinging to the twisted meaning-conferring role of despair that forms one’s identity around their suffering (Kierkegaard, 1980; Bernier, 2015; Kaftanski, 2021a; Hanson, 2024).

## Authenticity and well-being

Authenticity has attracted interest from scholars representing such diverse disciplines as philosophy, psychology, politics, and business to architecture, art, and tourism (Cohen, 1988; Taylor, 1991; Wang, 1999; Newman, 2019; Crawford et al., 2020). Authenticity has been defined and categorized in various ways in these disciplines. In their “Kinds of Authenticity” Newman and Smith (2016) present a multifaceted and wide-ranging review of types of authenticity in different fields of scholarship; they contribute their own “framework that organizes authenticity judgments along two core dimensions: the type of entity that is evaluated and the source of information that is consulted” (p. 609). The dominant approach to authenticity in psychology is along the lines of reading it in relation to such concepts as integrity and honesty (Peterson and Seligman, 2004),which are considered as features of “a character trait” of people whose internal lives are coherent with their private and public lives. Yet, for Peterson and Seligman, these words have slightly different meanings. “Honesty refers to factual truthfulness and interpersonal sincerity. Authenticity refers to emotional genuineness and psychological depth. Integrity refers to moral probity and self-unity”(Peterson and Seligman, 2004, p. 250).

Authenticity is overwhelmingly perceived as a fundamental aspect of well-being in counseling psychology (Yalom, 1980; Horney, 2013), despite its legitimate criticisms (Lasch, 1978). Pugh et al. (2017) define essentialist and existentialist approaches to authenticity in relation to a conception of selfhood. The essentialist approach to authenticity sees authentic life in one’s “self-discovery,” where an individual is authentic when they “live in accordance with [their] deep essence” (p. 641). The existentialist approach to authenticity emphasizes the efforts of an individual to self-create oneself unconstrained by social pressure, customs, and norms. Taking a quantitative approach to authenticity, Sutton (2020) defines it in relation to the “activity” of the self as “the activity of expressing one’s true self, making deliberate choices and taking responsibility for them” (p. 1).

The existentialist approaches to authenticity, which largely build on Kierkegaard’s work and legacy, indicate its role in enhancing well-being. In that tradition, Yalom (1980) points out the need to “to make things difficult again” in the effort to be authentic and hence increase one’s well-being. “Kierkegaard knew that man limited and diminished himself in order to avoid perception of the ‘terror, perdition and annihilation that dwell next door to any man,’” (Yalom, 1980, p. 111). Indeed, for Kierkegaard, authenticity requires one to face one’s shortcomings and reconcile one’s limitations, which although they cause suffering, are instrumental to a complete life. To be authentic means to be oneself—or to be more truthful to Kierkegaard’s vocabulary and ideas—to become oneself (Golomb, 1995). Becoming oneself is a process oriented toward an ideal of authenticity, which Kierkegaard understands as continuously choosing oneself, but also a conscious conditioning oneself through reflective meditation and deliberate habituation to pursuing realization of the ideals of oneself. This existential project of becoming oneself is an individual, hence deeply subjective, task; yet the validity of the principle of pursuing one’s true self is objective; hence it applies to everyone.

Negotiation of one’s ideal self and the actual self is built on Kierkegaard’s demand of reconciliation of human possibilities and limitations, which are famously stated in his view of existential-anthropological antinomies. Who we are for Kierkegaard is not just what we are made of (possibilities and limitations) but also about how we relate to our particular and universal contingencies (Rosfort, 2015). Kierkegaard’s vison of well-being shifts its focus from the area of morality and ethics understood in the Aristotelian sense of a good life to the rendering of well-being as based on one’s ability to relate to aspects of existence that are at times beyond our control (Kaftanski, 2021a). The three existential-anthropological antinomies central to the human life are: the infinite and the finite, the temporal and the eternal, freedom and necessity (Kierkegaard, 1980). Just like humans must not merely focus on the imaginative vision of their ideal self or on the lived life in the here and now, authentic existence holds both parts of the antinomies in check (Theunissen, 2005; Hanson, 2021a; Rudd, 2022). Neglecting freedom leads to a life overly regulated by our limitations. Such a life is the antithesis of well-being, namely despair (Hanson, 2022). Lacking the quality of possibility results in degenerating passivity of the individual’s self (Kierkegaard, 1980). On the other hand, the absence of the factor of necessity in one’s life leads them to what Kierkegaard understands as an abstract and alienated self that does not have a place in the world (Kierkegaard, 1980).

Many authors have sufficiently argued that the authentic self in Kierkegaard is essentially related to a curated project of its development (Westphal, 1996; Evans, 2006). Its blueprints are present in Kierkegaard’s famous stages of existence: esthetic, ethical, and religious (Hanson, 2016). They are essentially distinguished by an increase in the growth of awareness of a human being, by which Kierkegaard understands the capacity to reflectively think about oneself and others in the world. The ideal of the movement from the esthetic to the ethical to the religious constitutes a type of a formation of the self, which as such is historically based on the ideal of *Bildung* (Reindal, 2013). This formation is more than a training of moral dispositions of the good citizen, but also spiritual dispositions. Without the spiritual component, one is deprived of the full breadth of existence, because one is only limited to the temporal. The spiritual offers an extra motivation to commit to the project of one’s selfhood that avoids the perils of hyper-individualism.

## An integrated conceptualization of well-being

Because suffering is endemic to the process of personal growth and development for Kierkegaard, a subjective dimension to well-being must be posited. As objective goods are widely valued as a key component of meaningful living and as acknowledging the objective nature of human limitations is key to an authentic existence, so the proposed integrated conception incorporates an objective dimension as well. Essential to integrating the subjective and objective elements of human life, rather than positing them in opposition, is giving an account of how the subjective and objective dimensions constructively correlate in a proposed integrated conception of well-being. The correlation of the subjective and objective elements occurs through “identification” with the objective goods and pursuits one undertakes aligned with the actuality of one’s situation and circumstances in one’s envisioned ideal form.

Because individual selves develop in view of an imagined ideal, they do not in the first instance make decisions on the basis of principles or the fulfillment of social roles (Watts, 2017); rather, humans generate principles and adhere to the roles they adopt and the expectations those roles impose upon them in response to their aptitude for helping them bring their present actuality into line with their imagined ideal selves. Consequently, the process of personal development is one that proceeds by way of identification with or endorsement of the values, principles, and roles that individuals take to be conducive to the actualization of their envisioned ideals. On this integrated view of well-being, to value some end as worthwhile (even when it involves suffering) is not merely to subscribe to the reasons for valuing that end but to identify oneself with those reasons and thereby bind oneself to what one genuinely values and submits to judgment as to whether or not one is upholding their affirmed values.

As Anthony Rudd has argued, a person is not leading a meaningful life just by being able to tell an autobiographical story about that life; that person must identify with the protagonist of that autobiographical story (Rudd, 2008). An individual needs to identify with oneself. An example that is helpful to picture this process of self-identification is one presented in the work of Williams (1993). Williams presents a case of a bank clerk who hates his job, hence in an important sense he does not identify as a bank clerk. As Rudd argues, such a person “needs the money that he earns… so he wants to avoid getting sacked, and may even try to win promotion, but he does not care beyond that whether or not he does his job well” (Rudd, 1997, p. 73). Being bad as his jobs is not aligned with his “judgment on him *qua* person, only *qua* bank clerk, and he has no interest in being good at that” (Rudd, 1997, p. 73). Yet being a bank clerk can be instrumental to him for other reasons that are constitutive for him in a meaningful way. Being in fact a talented and passionate drummer in an aspiring rock band the man might even try to do the minimum required to keep that job and advance in it because the benefits of maintaining and progressing his situation allow him to pursue the musical activities he values and sees as constitutive of his ideal self. In that sense, what he identifies with is his being a drummer, not a bank clerk, and when he meets people at the club he tells them that what he is a drummer, not a bank clerk.

Crucial to this point is that the bank clerk identifies as a drummer in the sense that it is this capacity to which he is willing to be held to account and in this role that success or failure are of consequence to him and the extent to which he regards his life as an expression of his values. If he is a bad bank clerk that does not affect him as a person; if he is a bad drummer this is a grievous blow to his self-conception that he cannot accept with equanimity. What this paper claims arguing for an integrated conceptualization of well-being in relation to meaning in life is that it is not enough to notionally endorse a value or principle. One must stake themselves on it and thereby submit to its binding force, subjecting oneself to evaluation of either success or failure to live by what one has chosen. It is in this way that one must identify with their values and the reasons for them in their normative outlook, not just tell a story about these values or give them one’s provisional assent.

This view combines both objective and subjective aspects of well-being. To have a meaningful life on this view is not merely for that life to feature objectively valuable goods. It is to subjectively identify with those features or those features that one counts as genuinely valuable to themselves and according to which one is prepared to be judged for success or failure. As John Davenport (1998) explains, this task of identification is not simply a matter of adjudicating between one preference or perceived objective good over another:

To *identify* with desire A rather than B must involve more than merely having *another desire* to act on A. The higher-order volition is not merely a further desire or brute preference, but rather an attitude that essentially includes a non-arbitrary evaluation which itself involves “deciding what to think.” Identification is a process of *personally engaging* the whole self through a kind of reasoning, namely an “interested” or non-detached practical reasoning (Davenport, 1998, p. 365).

On this view of identification, which parallels the general argument that is made in this paper, a person must participate in conflicts within their own self, not merely be a spectator of them (Davenport, 1998). Similarly, Lynne McFall argues that “A person of integrity is willing to bear the consequences of her convictions, even when this is difficult…. A person whose only principle is ‘Seek my own pleasure’ is not a candidate for integrity because there is no possibility of conflict—between pleasure and principle—in which integrity could be lost” (McFall, 1987, p. 9).

What McFall is calling here “a person of integrity” is someone who embraces the suffering that may well be unavoidable in the process of working out the self one strives to become (McFall, 1987). This person is understood as a person of authenticity. Such an authentic individual is not tossed from one priority to another by a pseudo-commitment to their own fleeting preferences but is actively working out who they genuinely want to be, developing themselves toward their imagined ideal despite the difficulties entailed in becoming just such an authentic individual. Authenticity is this process of genuinely deciding what to think, what to value, and who to be. Authenticity depends crucially on identification, which demands more from the individual than notional support for a principle or halfhearted occupation of a social role, or a story about how one came to adopt such a principle or found oneself in a given role. Rather authenticity is a more wholehearted endorsement that entails readiness to participate in internal conflict and be judged for failure to uphold one’s own purported values.

This twofold conception of well-being developed in dialog with Kierkegaard is intentionally linked with Susan Wolf’s view of meaning in life that comprises both objective and subjective aspects.

A meaningful life must satisfy two criteria, suitably linked. First, there must be active engagement, and second, it must be engagement in (or with) projects of worth. A life is meaningless if it lacks active engagement with anything. A person who is bored or alienated from most of what she spends her life doing is one whose life can be said to lack meaning. Note that she may in fact be performing functions of worth… At the same time, someone who is actively engaged may also live a meaningless life, if the objects of her involvement are utterly worthless (Wolf, 1997, pp. 111–112).

Wolf’s account of meaning in life is isomorphic with the proposed account of well-being; in both cases this paper is postulating a subjective active engagement with projects of objective worth. The authentic person will subjectively be identified with the objective values and goods of their own existence. Similarly, on Wolf’s analysis, meaningful living depends not only on the pursuit of objective goods but also on the subject’s ability to actively engage with those goods.

Criticizing Wolf’s theory, Metz (2013) argues that meaning in life does not need a subjective aspect. Writing that he has “reason to doubt that any propositional attitude, positive or negative, is *necessarily* constitutive of one’s life being *somewhat* more meaningful,” Metz means that if something is meaningful, it is so without one’s subjective attitude toward it (p. 184). If what was argued above about the principle of identification is correct though, the precise character of the subjective dimension should be clearer as well as its importance to a comprehensive view of well-being. This paper argues that to identify with one’s life is to authentically appropriate it *as one’s own*. A life cannot be completely meaningful without such identification. This integrated conception of well-being, authenticity, and meaning in life can specify that the subjective dimension will not be either straightforwardly “positive or negative.” Rather, such an identification will require one to appropriate the actualities of one’s life, including those of suffering and “negativity,” as the cost of “positively” endorsing one’s existence as meaningfully one’s own.

## Conclusion

The integrated conceptualization of well-being argued in this paper positively identifies the role of suffering for human well-being by drawing on the notions of meaning in life and authenticity. This position on a conception of well-being challenges the dominant view of suffering in positive psychology as fundamentally negatively affecting human well-being, which essentially prescribes preventive or mitigating strategies to tackle suffering. The proposed integrated conceptualization of well-being suggests that alleviation of suffering should not be viewed as a blanket remedy for increasing well-being because, as has been argued, it often prevents individuals from considering opportunities for developing such well-being factors as meaning of life and authenticity. Drawing on the insights of the existentialist philosopher Søren Kierkegaard it has been demonstrated that suffering and negative affects are ineliminable from a meaningful life and indeed contribute to its meaningfulness. The integrated conceptualization of well-being successively addresses the subjective and objective dimensions of well-being emphasizing the role of the subjective in identification of objective good for a well-lived life. The hope of the authors of this article is that the argued integrated conceptualization of well-being will motivate scholars to develop new measures of well-being that consider suffering, meaning in life, authenticity, but also the subjective and objective elements of well-being.

## Author contributions

All authors listed have made a substantial, direct, and intellectual contribution to the work, and approved it for publication.

## Conflict of interest

The authors declare that the research was conducted in the absence of any commercial or financial relationships that could be construed as a potential conflict of interest.

## Publisher’s note

All claims expressed in this article are solely those of the authors and do not necessarily represent those of their affiliated organizations, or those of the publisher, the editors and the reviewers. Any product that may be evaluated in this article, or claim that may be made by its manufacturer, is not guaranteed or endorsed by the publisher.

## References

[ref1] Battista J. Almond R. (1973). The development of meaning in life. Psychiatry 36, 409–427. doi: 10.1080/00332747.1973.11023774 4756415

[ref2] Bernier M. (2015). The Task of Hope in Kierkegaard. Oxford: Oxford University Press.

[ref3] Bettis A. H. Coiro M. J. England J. Murphy L. K. Zelkowitz R. L. Dejardins L.. (2017). Comparison of two approaches to prevention of mental health problems in college students: enhancing coping and executive function skills. J. Am. Coll. Heal. 65, 313–322. doi: 10.1080/07448481.2017.1312411 28358274

[ref4] Bolier L. Haverman M. Westerhof G. J. Riper H. Smit F. Bohlmeijer E. (2013). Positive psychology interventions: a meta-analysis of randomized controlled studies. BMC Public Health 13:119. doi: 10.1186/1471-2458-13-119 23390882 PMC3599475

[ref5] Cassell E. J. (2002). “Compassion,” in Handbook of Positive Psychology. eds. Snyder C. R. Lopez S. J. (Oxford: Oxford University Press), 434–445.

[ref6] Cassell E. J. (2004). The Nature of Suffering and the Goals of Medicine. Oxford: Oxford University Press.

[ref7] CDC. (2018). Well-Being Concepts. Available at: https://www.cdc.gov/hrqol/wellbeing.htm#one (Accessed November 14, 2022).

[ref8] Cohen E. (1988). Authenticity and commoditization in tourism. Ann. Tour. Res. 15, 371–386. doi: 10.1016/0160-7383(88)90028-X

[ref9] Conley C. S. Durlak J. A. Kirsch A. C. (2015). A meta-analysis of universal mental health prevention programs for higher education students. Prev. Sci. 16, 487–507. doi: 10.1007/s11121-015-0543-1 25744536

[ref10] Cottingham J. (2003). On the Meaning of Life. New York and London: Routledge.

[ref11] Crawford J. A. Dawkins S. Martin A. Lewis G. (2020). Putting the leader back into authentic leadership: reconceptualising and rethinking leaders. Aust. J. Manag. 45, 114–133. doi: 10.1177/0312896219836460

[ref12] Davenport J. (1998). Piety, MacIntyre, and Kierkegaardian Choice. Faith Philos. 15, 352–365. doi: 10.5840/faithphil199815332

[ref13] Davis C. G. Nolen-Hoeksema S. Larson J. (1998). Making sense of loss and benefiting from the experience: two construals of meaning. J. Pers. Soc. Psychol. 75, 561–574. doi: 10.1037/0022-3514.75.2.561 9731325

[ref14] Edwards M. E. van Tongeren D. R. (2020). Meaning mediates the association between suffering and well-being. J. Posit. Psychol. 15, 722–733. doi: 10.1080/17439760.2019.1651890

[ref15] Elkin A. J. Rosch P. J. (1990). Promoting mental health at the workplace: the prevention side of stress management. Occup. Med. 5, 739–754. 2237702

[ref16] Evans S. C. (2006). Kierkegaard on Faith and the Self. Waco, TX: Baylor University Press.

[ref17] Fowers B. J. Richardson F. C. Slife B. D. (2017). Frailty, Suffering, and Vice: Flourishing in the Face of Human Limitations. Washington, DC: American Psychological Association.

[ref18] Fredrickson B. (2009). Positivity: Groundbreaking Research Reveals How to Embrace the Hidden Strength of Positive Emotions, Overcome Negativity, and Thrive. New York, NY: Crown Publishers.

[ref19] Glaw X. Kable A. Hazelton M. Inder K. (2016). Meaning in life and meaning of life in mental health care: an integrative literature review. Issues Ment. Health Nurs. 38, 1–13. doi: 10.1080/01612840.2016.1253804 27929687

[ref20] Golomb J. (1995). In Search of Authenticity: Existentialism from Kierkegaard to Camus. London and New York: Routledge.

[ref21] Hanson J. (2016). Kierkegaard and the Life of Faith: The Aesthetic, the Ethical, and the Religious in Fear and Trembling. Bloomington, IN: Indiana University Press.

[ref22] Hanson J. (2021a). Imagination, suffering, and perfection: a Kierkegaardian reflection on meaning in life. Hist. Philos. Quat. 38, 337–356. doi: 10.5406/21521026.38.4.03

[ref23] Hanson J. (2021b). Despair as a threat to meaning: Kierkegaard’s challenge to objectivist theories. Philosophies 6:92. doi: 10.3390/philosophies6040092

[ref24] Hanson J. (2022). “Despair the disease and faith the therapeutic cure,” in Kierkegaard’s the Sickness Unto Death: A Critical Guide. eds. Hanson J. Krishek S. (Cambridge: Cambridge University Press), 182–199.

[ref25] Hanson J. (2024). “Love as the antidote to despair,” in Cambridge Critical Guide to Works of Love (Forthcoming) eds. Hanson J. Kaftanski W. (Cambridge University Press).

[ref26] Haybron D. (2017). Well-being and virtue. J. Ethics Soc. Philos. 2, 1–28. doi: 10.26556/jesp.v2i2.21

[ref27] Heintzelman S. J. King L. A. (2013). On knowing more than we can tell: intuitive processes and the experience of meaning. J. Posit. Psychol. 8, 471–482. doi: 10.1080/17439760.2013.830758

[ref28] Hofmann B. (2022). Managing the moral expansion of medicine. BMC Med. Ethics 23:97. doi: 10.1186/s12910-022-00836-2 36138414 PMC9502962

[ref29] Horney K. (2013). Neurosis and Human Growth. London: Routledge.

[ref30] Kaftanski W. (2021a). Kierkegaard, Mimesis, and Modernity: A Study of imitation, Existence, and Affect. New York, NY and London: Routledge.

[ref31] Kaftanski W. (2021b). Introduction: imagination in Kierkegaard and beyond. Hist. Eur. Ideas 47, 405–413. doi: 10.1080/01916599.2020.1799552

[ref32] Kaftanski W. (2022). Imagination, mental representation, and moral agency: moral pointers in Kierkegaard and Ricoeur. Phenomenol. Cogn. Sci. doi: 10.1007/s11097-022-09813-x

[ref33] Kierkegaard S. (1980). The Sickness Unto Death. Princeton, NJ: Princeton University Press.

[ref34] Kierkegaard S. (1991). Practice in Christianity. Princeton, NJ: Princeton University Press.

[ref35] LaMontagne A. D. Keegel T. Vallance D. (2007). Protecting and promoting mental health in the workplace: developing a systems approach to job stress. Health Promot. J. Austr. 18, 221–228. doi: 10.1071/HE07221 18201165

[ref36] Lasch C. (1978). The Culture of Narcissism: American Life in an Age of Diminishing Expectations. New York, NY: W.W. Norton & Co.

[ref37] Martela F. Steger M. F. (2016). The three meanings of meaning in life: distinguishing coherence, purpose, and significance. J. Posit. Psychol. 11, 531–545. doi: 10.1080/17439760.2015.1137623

[ref38] McFall L. (1987). Integrity. Ethics 98, 5–20.

[ref39] Metz T. (2013). Meaning in Life. Oxford: Oxford University Press.

[ref41] Morrison E. Burke G. Greene L. (2007). Meaning in motivation: does your organization need an inner life? J. Health Hum. Serv. Adm. 30, 98–115. 17557698

[ref42] Newman G. E. (2019). The psychology of authenticity. Rev. Gen. Psychol. 23, 8–18. doi: 10.1037/gpr0000158

[ref43] Newman G. E. Smith R. K. (2016). Kinds of authenticity. Philos. Compass 11, 609–618. doi: 10.1111/phc3.12343

[ref44] Park C. L. (2008). Testing the meaning making model of coping with loss. J. Soc. Clin. Psychol. 27, 970–994. doi: 10.1521/jscp.2008.27.9.970

[ref45] Park C. L. Edmondson D. Fenster J. R. Blank T. O. (2008). Meaning making and psychological adjustment following cancer: the mediating roles of growth, life meaning, and restored just-world beliefs. J. Consult. Clin. Psychol. 76, 863–875. doi: 10.1037/a0013348 18837603

[ref46] Peterson C. Seligman M. (2004). Character Strengths and Virtues. Oxford: Oxford University Press.

[ref47] Pugh J. Maslen H. Savulescu J. (2017). Deep brain stimulation, authenticity and value. Camb. Q. Healthc. Ethics 26, 640–657. doi: 10.1017/S0963180117000147 28937346 PMC5658726

[ref48] Quick J. Henderson D. (2016). Occupational stress: preventing suffering, enhancing wellbeing. Int. J. Environ. Res. Public Health 13:459. doi: 10.3390/ijerph13050459 27136575 PMC4881084

[ref49] Reindal S. M. (2013). Bildung, the Bologna process and Kierkegaard’s concept of subjective thinking. Stud. Philos. Educ. 32, 533–549. doi: 10.1007/s11217-012-9344-1

[ref50] Rosfort R. (2015). “Kierkegaard’s conception of psychology,” in A Companion to Kierkegaard. ed. Stewart J. (Chichester: Blackwell Publishing Ltd.), 451–467.

[ref51] Rudd A. (1997). Kierkegaard and the Limits of the Ethical. Oxford: Oxford University Press.

[ref52] Rudd A. (2008). Reason in ethics revisited. Kierkegaard Stud. Yearb. 2008, 179–199. doi: 10.1515/9783110205350.1.179

[ref53] Rudd A. (2022). “Kierkegaard on the self and the modern debate on selfhood,” in Kierkegaard’s the Sickness unto Death: A Critical Guide. eds. Hanson J. Krishek S. (Cambridge: Cambridge University Press), 42–60.

[ref54] Sacks J. (2007). To Heal a Fractured World: The Ethics of Responsibility. New York, NY: Schocken Books.

[ref57] Seligman M. (2012a). Flourish: A Visionary New Understanding of Happiness and Well-Being New York, NY: Atria.

[ref58] Seligman M. (2012b). “Flourish: positive psychology and positive interventions,” in The Tanner Lectures on Human Values, vol. 31 (University of Utah Press), 231–243.

[ref59] Seligman M. (2018). PERMA and the building blocks of well-being. J. Posit. Psychol. 13, 333–335. doi: 10.1080/17439760.2018.1437466

[ref60] Simons G. Baldwin D. S. (2021). A critical review of the definition of ‘wellbeing’ for doctors and their patients in a post Covid-19 era. Int. J. Soc. Psychiatry 67, 984–991. doi: 10.1177/00207640211032259 34240644 PMC8592098

[ref61] Steger M. F. (2009). “Meaning in life,” in Oxford Handbook of Positive Psychology. 2nd eds. Lopez S. J. Snyde C. R. (Oxford University Press), 679–687.

[ref62] Stjernswärd J. Foley K. M. Ferris F. D. (2007). The public health strategy for palliative care. J. Pain Symptom Manag. 33, 486–493. doi: 10.1016/j.jpainsymman.2007.02.016 17482035

[ref63] Sutton A. (2020). Living the good life: a meta-analysis of authenticity, well-being and engagement. Personal. Individ. Differ. 153:109645. doi: 10.1016/j.paid.2019.109645

[ref64] Taylor C. (1991). The Ethics of Authenticity. Cambridge, MA: Harvard University Press.

[ref65] Theunissen M. (2005). Kierkegaard’s Concept of Despair. Princeton, NJ: Princeton University Press.

[ref66] Wang N. (1999). Rethinking authenticity in tourism experience. Ann. Tour. Res. 26, 349–370. doi: 10.1016/S0160-7383(98)00103-0

[ref67] Watkin J. (2001). Historical Dictionary of Kierkegaard’s Philosophy. Lanham, MD: The Scarecrow Press.

[ref68] Watts D. (2017). Kierkegaard, repetition and ethical Constancy. Philos. Investig. 40, 414–439. doi: 10.1111/phin.12169

[ref69] Westphal M. (1996). Becoming a Self: A Reading of Kierkegaard’s “Concluding Unscientific Postscript”. West Lafayette, IN: Purdue University Press.

[ref70] WHO. (2022). Health and Well-Being. Available at: https://www.who.int/data/gho/data/major-themes/health-and-well-being (Accessed November 14, 2022).

[ref71] Williams B. (1993). Morality: An Introduction to Ethics. Cambridge: Cambridge University Press.

[ref72] Wolf S. (1997). Happiness and meaning: two aspects of the good life. Soc. Philos. Policy 14, 207–225. doi: 10.1017/S0265052500001734

[ref73] Wong P. T. P. (2011). Positive psychology 2.0: towards a balanced interactive model of the good life. Can. Psychol. 52, 69–81. doi: 10.1037/a0022511

[ref74] Wong P. T. P. (2017). Meaning-centered approach to research and therapy, second wave positive psychology, and the future of humanistic psychology. Humanist. Psychol. 45, 207–216. doi: 10.1037/hum0000062

[ref75] Wong P. T. P. Bowers V. (2018). “Mature happiness and global wellbeing in difficult times,” in Scientific Concepts Behind Happiness, Kindness, and Empathy in Contemporary Society. ed. Silton N. R. (Hershey, PA: IGI Global), 112–134.

[ref76] Wong P. T. P. Mayer C.-H. Arslan G. (2021). Editorial: COVID-19 and existential positive psychology (PP2.0): the new science of self-transcendence. Front. Psychol. 12:800308. doi: 10.3389/fpsyg.2021.800308 34956025 PMC8699172

[ref77] Yalom I. D. (1980). Existential Psychotherapy. New York, NY: Basic Books.

